# The characteristics of extrachromosomal circular DNA in patients with end-stage renal disease

**DOI:** 10.1186/s40001-023-01064-z

**Published:** 2023-03-27

**Authors:** Yue Peng, Yixi Li, Wei Zhang, Yu ShangGuan, Ting Xie, Kang Wang, Jing Qiu, Wenjun Pu, Biying Hu, Xinzhou Zhang, Lianghong Yin, Donge Tang, Yong Dai

**Affiliations:** 1 Clinical Medical Research Center, Guangdong Provincial Engineering Research Center of Autoimmune Disease Precision Medicine, Shenzhen Engineering Research Center of Autoimmune Disease, The Second Clinical Medical College of Jinan University, Shenzhen People’s Hospital, Jinan University, Shenzhen, China; 2 Institute of Nephrology and Blood Purification, The First Affiliated Hospital of Jinan University, Jinan University, Guangzhou, China; 3 Key Renal Laboratory of Shenzhen, Department of Nephrology, Shenzhen People’s Hospital, The Second Clinical Medical College of Jinan University, Shenzhen, 518020 Guangdong China; 4 Guangzhou Enttxs Medical Products Co., Ltd. P.R. Guangzhou, Guangzhou, China; 5 Department of Pathology, The 924th Hospital of the Chinese People’s Liberation Army Joint Logistic Support Force, Guangxi Key Laboratory of Metabolic Diseases Research, Guilin, 541002 Guangxi China

**Keywords:** eccDNA, Chronic kidney disease, End-stage renal disease, High-throughput sequencing, Circle map

## Abstract

**Background:**

End-stage renal disease (ESRD) is the final stage of chronic kidney disease (CKD). In addition to the structurally intact chromosome genomic DNA, there is a double-stranded circular DNA called extrachromosomal circular DNA (eccDNA), which is thought to be involved in the epigenetic regulation of human disease. However, the features of eccDNA in ESRD patients are barely known. In this study, we identified eccDNA from ESRD patients and healthy people, as well as revealed the characteristics of eccDNA in patients with ESRD.

**Methods:**

Using the high-throughput Circle-Sequencing technique, we examined the eccDNA in peripheral blood mononuclear cells (PBMCs) from healthy people (NC) (*n* = 12) and ESRD patients (*n* = 16). We analyzed the length distribution, genome elements, and motifs feature of eccDNA in ESRD patients. Then, after identifying the specific eccDNA in ESRD patients, we explored the potential functions of the target genes of the specific eccDNA. Finally, we investigated the probable hub eccDNA using algorithms.

**Results:**

In total, 14,431 and 11,324 eccDNAs were found in the ESRD and NC groups, respectively, with sizes ranging from 0.01 kb to 60 kb at most. Additionally, the ESRD group had a greater distribution of eccDNA on chromosomes 4, 11, 13, and 20. In two groups, we also discovered several motifs of specific eccDNAs. Furthermore, we identified 13,715 specific eccDNAs in the ESRD group and 10,585 specific eccDNAs in the NC group, both of which were largely annotated as mRNA catalog. Pathway studies using Gene Ontology (GO) and the Kyoto Encyclopedia of Genes and Genomes (KEGG) showed that the specific eccDNA in ESRD was markedly enriched in cell junction and communication pathways. Furthermore, we identified potentially 20 hub eccDNA-targeting genes from all ESRD-specific eccDNA-targeting genes. Also, we found that 39 eccDNA-targeting genes were associated with ESRD, and some of these eccDNAs may be related to the pathogenesis of ESRD.

**Conclusions:**

Our findings revealed the characteristics of eccDNA in ESRD patients and discovered potentially hub and ESRD-relevant eccDNA-targeting genes, suggesting a novel probable mechanism of ESRD.

**Supplementary Information:**

The online version contains supplementary material available at 10.1186/s40001-023-01064-z.

## Background

End-stage renal disease (ESRD) is the final stage of chronic kidney disease (CKD), characterized by a glomerular filtration rate (GFR) of less than 15 ml/min/1.73m^2^, as well as structural and functional deterioration for at least 3 months [1]. According to the recent studies, diabetes and hypertension are the primary causes of ESRD in developed countries [2], whereas the transition from glomerulonephritis to metabolic kidney disorders has become the leading cause in low- and middle-income nations [3, 4]. The prevalence of ESRD has risen considerably as diabetes and hypertension have become more prevalent [5, 6]. Currently, kidney replacement therapy (KRT), which includes hemodialysis, peritoneal dialysis, and kidney transplantation, is the most common treatment for ESRD. Global KRT usage is predicted to reach 5.4 million by 2030, with Asia seeing the most growth [7]. Cardiovascular illnesses, uremia, volume overload, malnutrition, uncontrolled hypertension, cancer, severe infections, and dialysis-related complications are all major causes of death in people with ESRD [8–10]. ESRD has become a global concern since it would significantly diminish life quality and increase financial cost on families and society.

Yasuo Hotta and Alix Bassel discovered extrachromosomal circular DNA (eccDNA) in pig sperm and wheat nuclei in 1964 [11]. EccDNA is a double-stranded circular DNA that exists in addition to the structurally complete chromosome genomic DNA. Following that, eccDNAs were discovered in human tumor cells [12] and practically all organisms [13, 14]. EccDNAs have been classified as micro DNA (100–400 bp) [15], small polydispersed circular DNAs (spcDNAs, 100 bp–10 kb) [16], mitochondrial DNAs (mtDNAs, 16 kb) [17], and double-minutes (DMs, 100 kb–3 Mb) [17, 18] based on their length and features. Previous research has linked eccDNA to genomic rearrangement, cell apoptosis, episome enlargement, translocation, and amplification [19–22]. Several studies have proclaimed that eccDNA potentially play an important role in disorders involving epigenetic modulation, such as cancer [23]. EccDNA can cause gene deletion, mutation, duplication, or amplification, resulting in genetic heterogeneity and adaptive evolution between cells. Recent research by lv [24] examined the physical characteristics of eccDNA, such as length, GC content, and motif signature, etc. and miRNA in urine from CKD patients, and found that the eccDNA count in the CKD group was higher than it was in the healthy group. Although preliminary study on the properties of eccDNA in CKD patients revealed certain differences between the patients and healthy individuals, there is still a dearth of knowledge regarding the role and probable mechanism of eccDNA in CKD, particularly in ESRD patients, which we intended to focus on.

In this study, we used a high-throughput approach, namely Circle-sequencing, to collect and identify eccDNAs from peripheral blood mononuclear cells (PBMCs) of ESRD patients (*n* = 16) and healthy people (*n* = 12). We detected 14,431 and 11,324 eccDNAs in the ESRD and NC groups, respectively, with a large range of eccDNA sizes. Furthermore, we identified that the specific eccDNA in ESRD was markedly enriched in cell junction and communication pathways. Out of all the eccDNA-targeting genes specific to ESRD, we discovered potentially 20 hub genes. Additionally, we found that eccDNA-targeting genes, including CCL2, CCR2, MYH9, and IL10, were critical in the development of ESRD, indicating that these eccDNAs had novel biological roles for ESRD patients.

## Methods

### Participants

From January 2020 to January 2021, 16 ESRD patients and 12 healthy individuals participated in this study at Shenzhen People’s Hospital (Shenzhen, China). This study’s ESRD diagnostic criteria were based on the CKD diagnostic and grading guideline of the 2012 Kidney Disease: Improving Global Outcomes (KDIGO) and Kidney Disease Outcomes Quality Initiative (KDOQI) guideline [25], with the following inclusion criteria: (1) age ≥ 18 years; (2) abnormalities of kidney structure present for > 3 months, including: abnormalities detected by imaging or histology, electrolyte and other abnormalities due to tubular disorders; (3) abnormalities of kidney function (GFR < 15 mL/min/1.73m^2^), present for > 3 months; (4) maintenance hemodialysis three times a week for at least 6 months; (5) without acute or chronic infection; (6) without malignant tumor; (7) without no serious complications of important organs, including cardiovascular, hepatic, pulmonary, or brain. The individuals selected in the normal control (NC) group were from the Physical Examination Center of Shenzhen People's Hospital (Shenzhen, China), which had no relation in birth with ESRD patients, with the inclusion and exclusion criteria as follows: (1) age ≥ 18 years; (2) no clinical or laboratory evidence for renal diseases; (3) without acute or chronic infection; (4) without malignant tumor; (5) without critical basic diseases, including urinary, cardiovascular, hepatic, pulmonary, or brain related issues. The ESRD group was studied using demographic profiles, such as age, gender, hemodialysis duration, hypertension (HTN), diabetic nephropathy (DN), chronic glomerulonephritis (CGN), and lupus nephropathy (LN), as well as laboratory data such as albumin, hemoglobin (Hb), neutrophil percentage, estimated glomerular filtration rate (eGFR), serum creatinine (Scr), blood urea nitrogen (BUN), and parathyroid hormone (PTH) as independent variables. Shenzhen People's Hospital Ethics Committee approved the study, which was carried out in accordance with the Declaration of Helsinki. All participants signed their informed consent.

### PBMC extraction

A heparinized vacuum container was used to collect each 10 mL blood sample from the ESRD and NC groups. PBMCs were isolated from blood samples using Ficoll-Hypaque Solution (GE Healthcare, Marlborough, MA) by density gradient centrifugation at 1200 rpm for 3 min at room temperature, and lysed using TRIzol reagent (Invitrogen; Thermo Fisher Scientific, Inc.) after standing at 4 °C for 20 min [26, 27]. The suspension was then transferred to a new tube and centrifuged again (2000 rpm for 15 min). Finally, the supernatant was immediately collected, and the PBMCs were kept in a refrigerator at − 80 °C.

### Total DNA isolation

To rupture plasma cell membranes and eliminate DNase and RNase, total DNA was isolated from PBMCs in lysis buffer containing L1 suspension solution (A&A Biotechnology) supplemented with Proteinase K [28]. Samples were incubated in the aforementioned lysate for 16 to 24 h at 50 °C and 700 rpm, until the suspension was homogeneous and acellular mass, before being chilled to room temperature. DNA samples were purified and enhanced using the Plasmid Mini AX Kit’s instructions for column chromatography (A&A Biotechnology).

### Linear chromosomal DNA digestion

The purified DNA was treated with cutting endonuclease to facilitate specific digestion of linear DNA and part of mtDNA by exonuclease. The samples were incubated for 16 h at 37 °C, then the endonuclease was heat inactivated for 5 min at 80 °C. Exonuclease (Plasmid-Safe ATP-Dependent DNase kit, Epicentre) was used to digest linear single-stranded and double-stranded DNA. After removal of the above DNA, samples from the exonuclease-treated solution were confirmed to eliminate chromosomal linear DNA and mtDNA by quantitative polymerase chain reaction (qPCR) using gene COX5B and Human mt separately. The exonuclease solution was then heat inactivated for 30 min at 70 °C and the digested eccDNA was purified by magnetic beads (Agencourt AMPure XP beads).

### eccDNA amplification

Enriched and purified eccDNA was subjected to rolling circle amplification with the REPLI-g Mini Kit (Qiagen, 150023), and the amplified product was purified with AMPure XP beads (Beckman, A63880).

### High-throughput sequencing

With a focused ultrasonicator (biorupter), the rolling circle amplification products were ultrasonically sheared to roughly 200–300 bp. The NEBNext Ultra DNA Library Prep Kit for Illumina was used to recover the fragmented products and build libraries. Finally, the libraries were sent to an Illumina Novaseq 6000 system for sequencing. Paired-end 150 sequencing approach (PE150).

### Raw sequencing data analysis

After the library check was qualified, raw sequencing data was generated from the Illumina NovaSeq 6000 system. Trimmomatic software [29] filtered raw data to remove adaptor and low-quality reads, and default reference values were utilized for the key parameters. The clean reads produced from the raw reads by eliminating the adaptor sequences were then aligned to reference genome sequences (hg38_gencode) using the BWA tool after raw reads and clean reads quality testing. The reads aligned to the gene were used for subsequent identification and analysis of circular DNA. The circular DNA was detected by the Circle-Map [30, 31], and the split was used for screening eccDNA. Each eccDNA contained at least 1 split read (split ≥ 1), which was chosen to be further analyzed.

### Gene annotation and functional analyses

The gene annotation of eccDNA was based on BEDTools [32]. The HOMER's findMotifsGenome.pl tool was used for motif analysis. Gene Ontology (GO) analysis of the specifically expressed genes on eccDNAs was derived from Database for Annotation, Visualization, and Integrated Discover (DAVID, https://david.ncifcrf.gov/tools.jsp). Kyoto Encyclopedia of Genes and Genomes (KEGG) pathway was utilized to describe specifically expressed gene pathways on the basis of the DAVID database as well. Fisher exact test was performed to detect the overlap between the GO/KEGG annotation lists. *P* < 0.05 was considered to be statistically significant of GO terms/KEGG pathways. When the eccDNAs were classified into distinct types of RNAs, including miRNA, lncRNA, mRNA, pseudogene, and other types of genes (others), if multiple sorts were annotated, eccDNAs were classified as multipleType. For the sake of obtaining known and predicted functional associations between specific expression genes on eccDNAs, STRING database (https://cn.string-db.org/) was used. Furthermore, the Molecular Complex Detection (MCODE) in Cytoscape was used to inspect the modules of the gene–gene interaction (GGI) network (degree cutoff = 2, node score cutoff = 0.2, k-core = 2, and max. Depth = 100). The cytoHubba plugin in Cytoscape was used to identify the top 20 hub genes in network GGI by MCC method. The known genes associated with ESRD were searched in the Phenopedia database (https://phgkb.cdc.gov/PHGKB/startPagePhenoPedia.action) and Disgenet database (https://www.disgenet.org). The Metascape database (https://metascape.org) was conducted for pathway analyses of ESRD-related genes.

### Statistical analysis

Categorical variables were expressed as frequency and percentage (%), and compared by the *χ*^2^ test between groups. Normally distributed numerical variables were described as mean ± SD and compared by the Student’s *t* test between groups, while non-normally distributed numerical variables were presented as median [P_25_, P_75_].

## Results

### Basic information of participants

The clinical and demographic data of ESRD patients and healthy volunteers was shown in Table 1. The control group had the matched gender with ESRD group (*P* > 0.05), while the age of the controls meeting the grouping conditions was younger. By comparison, each laboratory profile of patients with ESRD was visibly aberrant, while hemoglobin, neutrophil percentage, serum creatinine, and blood urea nitrogen of the NC group were in the normal range and had a significant difference from patients with ESRD (*P* < 0.05).

**Table 1:** The demographic and laboratory data in ESRD and NC groups

Variables	ESRD (*n* = 16)	NC (*n* = 12)	*P* value
Age, years (± SD)	45 ± 10	33 ± 13	0.007
Male, *n* (%)	8 (50.00)	4 (33.33)	0.3778
HTN, *n* (%)	14 (87.50)	/	/
DN, *n* (%)	4 (25.00)	/	/
CGN, *n* (%)	10 (62.50)	/	/
LN, *n* (%)	2 (12.50)	/	/
Duration of hemodialysis, years (M [P_25_, P_75_])	4 (2.75, 5.00)	/	/
Hb, g/dL (± SD)	93.63 ± 16.31	137.00 ± 14.72	< 0.0001
Neutrophil percentage, % (± SD)	66.29 ± 7.46	56.22 ± 10.16	0.0076
Scr, µmol/L (± SD)	1027.76 ± 367.35	71.97 ± 13.75	< 0.0001
BUN, mmol/L (± SD)	21.81 ± 7.62	3.95 ± 0.73	< 0.0001
eGFR, mL/min/1.73m^2^ (± SD)	4.93 ± 2.99	/	/
Albumin, g/dL (± SD)	39.28 ± 6.57	/	/
PTH, pg/mL (± SD)	381.57 ± 300.49	/	/

ESRD, end-stage renal disease; NC, normal control; HTN, hypertension; DN, diabetes nephropathy; CGN, chronic glomerulonephritis; LN, lupus nephritis; Hb, hemoglobin; Scr, serum creatinine; BUN, blood urea nitrogen; eGFR, estimated glomerular filtration rate; PTH, parathyroid hormone

### The quality control of circle sequencing

After sequencing, we yielded 159,339,104, and 133,069,578 raw reads from the NC and ESRD groups, respectively. Then, using Trimmomatic to remove adaptor and low-quality sequences, we obtained 156,391,184 and 130,528,434 clean reads from the NC and ESRD samples, respectively. Following that, we found that the quality values Q20 and Q30 in two groups were both greater than 90% as analyzed through FastQC, which suggested strong sequencing accuracy. Subsequently, we discovered that the unique mapped rate was both over 80%, indicating good sequencing quality. The data of quality control were listed in Table 2.

**Table 2:** The quality control data of circle-sequencing

Sample	Raw reads	Clean reads	Q20 (%)	Q30 (%)	Unique mapped rate (%)
NC	159,339,104	156,391,184	98.32	94.61	88.45
ESRD	133,069,578	130,528,434	98.35	94.68	84.53

### The length ranges and genomic distribution of eccDNA

**Fig. 1:**
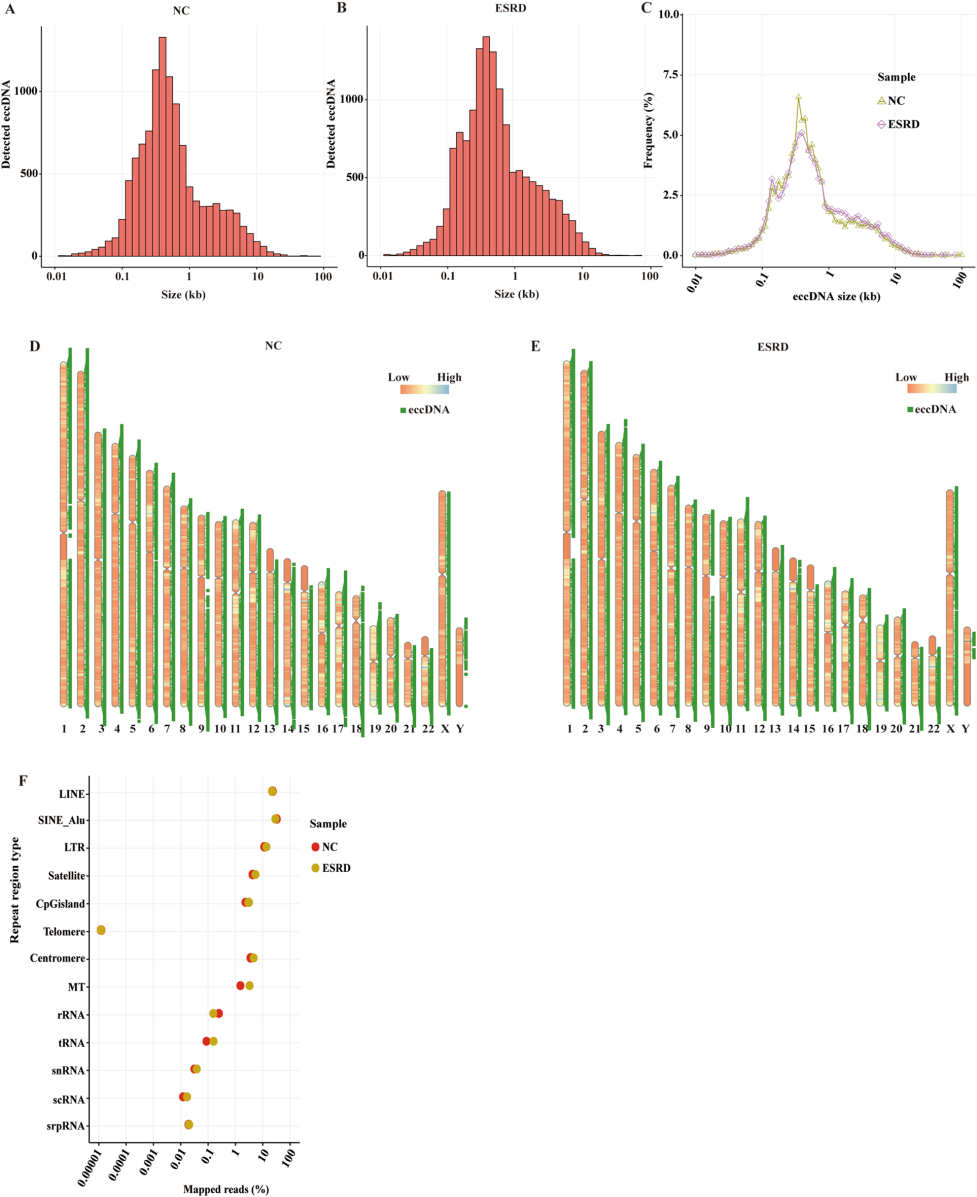
EccDNA sizes, distribution, repetitive elements, and catalogs. The distribution of eccDNA sizes (< 100 kb) in the NC (**A**) and ESRD (**B**) groups. Plots of the size distributions (< 100 kb) of ESRD- and NC-derived eccDNA (**C**). Distribution of eccDNA on the chromosome genome in NC (**D**) and ESRD (**E**) groups. Repetitive regions from general mapped reads for ESRD- and NC-derived eccDNA samples (**F**)

We used the Circle-Map software under the screening condition with split reads ≥ 1 to detect eccDNA from similar pair areas of the human genome. As a result, we identified more than 10,000 distinct eccDNAs in the ESRD (*n* = 14,431) and NC groups (*n* = 11,324), respectively, and eccDNA ranged from 0.01 KB to 60 KB at most (Fig. 1A, B). In the NC group, there were nine eccDNAs greater than 1 MB, with the longest being 218 Mb. There were 15 eccDNAs larger than 1 MB in the ESRD sample, with the largest being 88 MB. The fraction of eccDNA in ESRD was higher than that in NC in most length ranges, but in the 0.3 kb to 1 kb range, NC's proportion was higher than ESRD's (Fig. 1C). As was previously reported, the full range of circular DNAs was included in our study [23]. We found that ESRD patients' eccDNAs were less distributed than the NC group's on chromosomes 2, 17, and 19 and sex chromosome Y, but that healthy individuals' eccDNAs were less disseminated than ESRD samples on chromosomes 4, 11, 13, and 20 (Fig. 1D, E).

In previous studies, it has been known that eccDNA consists of all types of elements, including repetitive genomic elements, and the repetitive regions exist in proportions to the genome [33]. In our study, we also found that a large number of reads mapped to long interspersed nuclear elements (LINE), short interspersed nuclear Alu (SINE-Alu), long terminal repeat (LTR), satellite, CpG island, telomere, centromere, mitochondria (MT), ribosomal RNA (rRNA), transfer RNA (tRNA), small nuclear RNA (snRNA), single-cell RNA (scRNA), and signal recognition particle RNA (srpRNA), implying the eccDNAs derived from all types of repetitive sequences on genome (Fig. 1F). With the University of California Santa Cruz database (UCSC, http://genome.ucsc.edu), we discovered that the eccDNA reads in LINE (18.2% of ESRD, 18.5% of NC), SINE-Alu (23.7% of ESRD, 26.1% of NC), and LTR (11.2% of ESRD, 9.6% of NC) were obviously enriched, but reads in telomere were fewer. In the ESRD group, reads in MT (2.7%) and tRNA (0.1%) regions were apparently more abundant than in the NC group (1.3% of MT, 0.07% of tRNA). On the contrary, reads in rRNA regions in ESRD (0.1%) were fewer than in NC (0.2%).

### The identified motifs on both sides of eccDNA junctions

**Fig.2:**
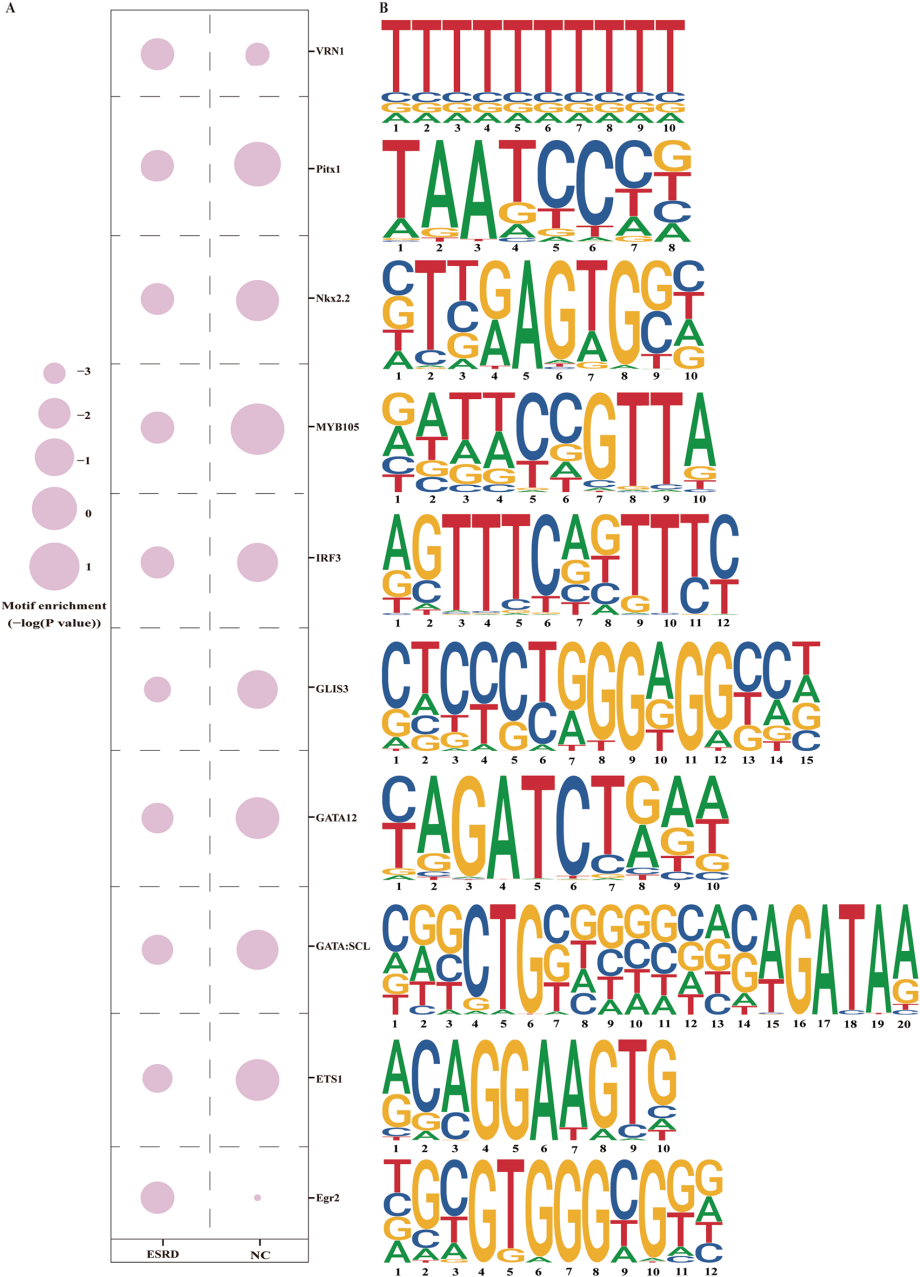
Identification of motifs and transcription factors. Enrichment scores of top 10 predicted transcription factors binding motifs in ESRD group and compared to NC group (**A**). The top 10 transcription factors related motif sequences (**B**)

We probed recurring motifs that appeared on both sides of the eccDNA junction site and their corresponding transcription factors (TFs) to explore the effect of TFs on eccDNA functions. After identifying the junction sites of eccDNA, we retrieved the DNA sequences that were enlarged to 200 bp in the upstream and downstream directions of eccDNA coordinate start and end, respectively. The start and end junction locations are thought to be the consequence of chromosomal DNA sequences being cut off to generate eccDNA [34]. According to 37,874 and 39,462 genome DNA sequences in the NC and ESRD groups, separately, findMotifsGenome.pl tool of Homer software was used to identify the known motifs and relevant TFs. As a result, we discovered 976 motifs in total, 20 and 38 of which were substantially connected to the TFs (*P* value ≤ 0.01) in the ESRD and NC groups, respectively. The 20 motifs in ESRD patients’ eccDNA were collectively listed in Table 3. The enrichment scores of the top 10 TF binding motifs were also recorded with a comparison of the NC group (Fig. 2A, B).

**Table 3:** Identification of motif patterns in ESRD

Motif sequences	*P* value
CTCCCTGGGAGGCCN	1e−5
ACAGGAAGTG	1e−4
CRGCTGBNGNSNNSAGATAA	1e−3
YAGATCTRAW	1e−3
TAATCCCN	1e−2
BTBRAGTGSN	1e−2
TTTTTTTTTT	1e−2
RATWCCGTTA	1e−2
AGTTTCAKTTTC	1e−2
NGCGTGGGCGGR	1e−2
AAAAAAAAAA	1e−2
AVCCGGAAGT	1e−2
AAACMATTAN	1e−2
RSCACTYRAG	1e−2
RGGATTAR	1e−2
CDCCGCCGTC	1e−2
ATGCATAATTCA	1e−2
BCWGATTCAATCAAN	1e−2
AAAGRGGAAGTG	1e−2
TWCCHWATWDGGAAA	1e−2

N, degenerate bases of A/T/C/G; R, degenerate bases of A/G; B, degenerate bases of T/C/G; S, degenerate bases of C/G; Y, degenerate bases of T/C; W, degenerate bases of A/T

### Functional investigations of target genes of ESRD-specific eccDNAs

**Fig. 3:**
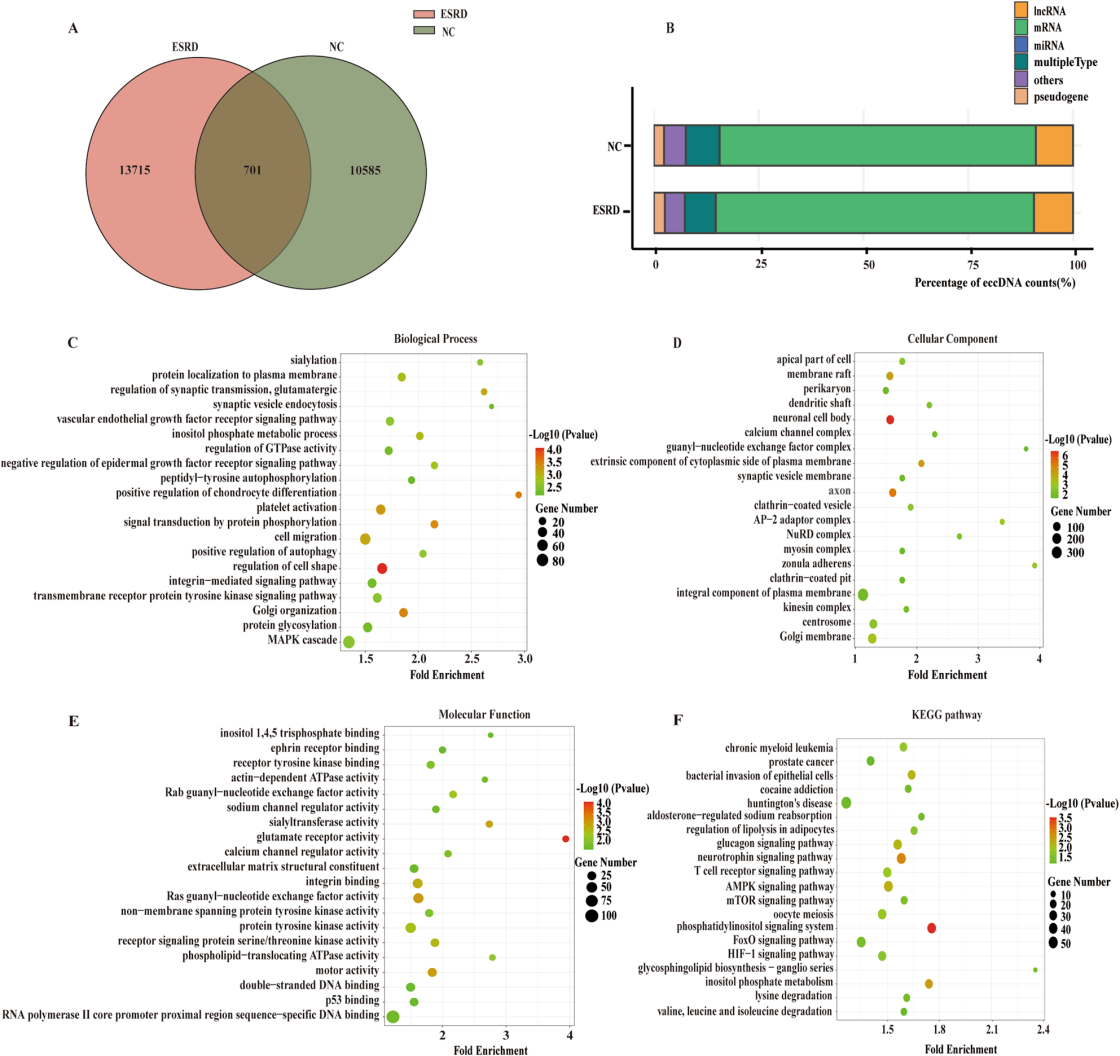
Specifically expressed eccDNA number, catalogs, and GO/KEGG functional enrichment bubble plots in patients with ESRD compared. Venn diagram shows the number of commonly and specifically expressed eccDNA in the ESRD group and NC group (**A**). The catalog distribution of specifically expressed eccDNA in NC and ESRD groups (**B**). The GO classifications of specifically expressed eccDNA in patients with ESRD in the biological processes (**C**), the cellular component (**D**), and molecular function (**E**). The specifically eccDNA in patients with ESRD in the KEGG functional enrichment (**F**)

To define the specific eccDNA in ESRD patients which may have an effect on the occurrence of ESRD, we analyzed the eccDNAs detected in ESRD patients and healthy people using Venn analysis. To avoid influencing the results, 1 Mb of eccDNA was left out of the analysis (1 Mb in length would interfere with the intersection analysis). The result revealed 13,715 specific eccDNAs in the ESRD group and 10,585 in the NC groups, respectively (Fig. 3A). After that, to probe the probable functions of these peculiar eccDNAs in the development of ESRD, we performed enrichment analysis of the target genes of the eccDNA using BEDtools. Then, we classified the eccDNAs into different modules based on the catalogs of their targeting genes. Eventually, there was no significant difference in quantity between specific eccDNAs in ESRD patients and the NC group among the diverse modules. Most part of the specifically expressed eccDNA was categorized as mRNA (Fig. 3B).

Furthermore, we conducted GO and KEGG analysis of these specific eccDNAs’ targeting genes. Compared with the NC group, genes on eccDNAs in ESRD patients were particularly enriched in the biological processes, namely regulation of cell shape, positive regulation of chondrocyte differentiation, and Golgi organization (Fig. 3C), the cellular components, such as neuronal cell body, axon, and extrinsic component of cytoplasmic side of plasma membrane (Fig. 3D), and the molecular functions, which were glutamate receptor activity, Ras guanyl-nucleotide exchange factor activity, and motor activity (Fig. 3E). Meanwhile, the KEGG analysis highlighted three pathways, including phosphatidylinositol signaling system, neurotrophin signaling pathway, and inositol phosphate metabolism (Fig. 3F). According to the aforementioned findings, eccDNAs might be involved in the pathways, such as chondrocyte differentiation, Golgi function, and phosphatidylinositol signaling, to promote the development and progression of ESRD.

### The potentially hub eccDNA in ESRD

**Fig. 4:**
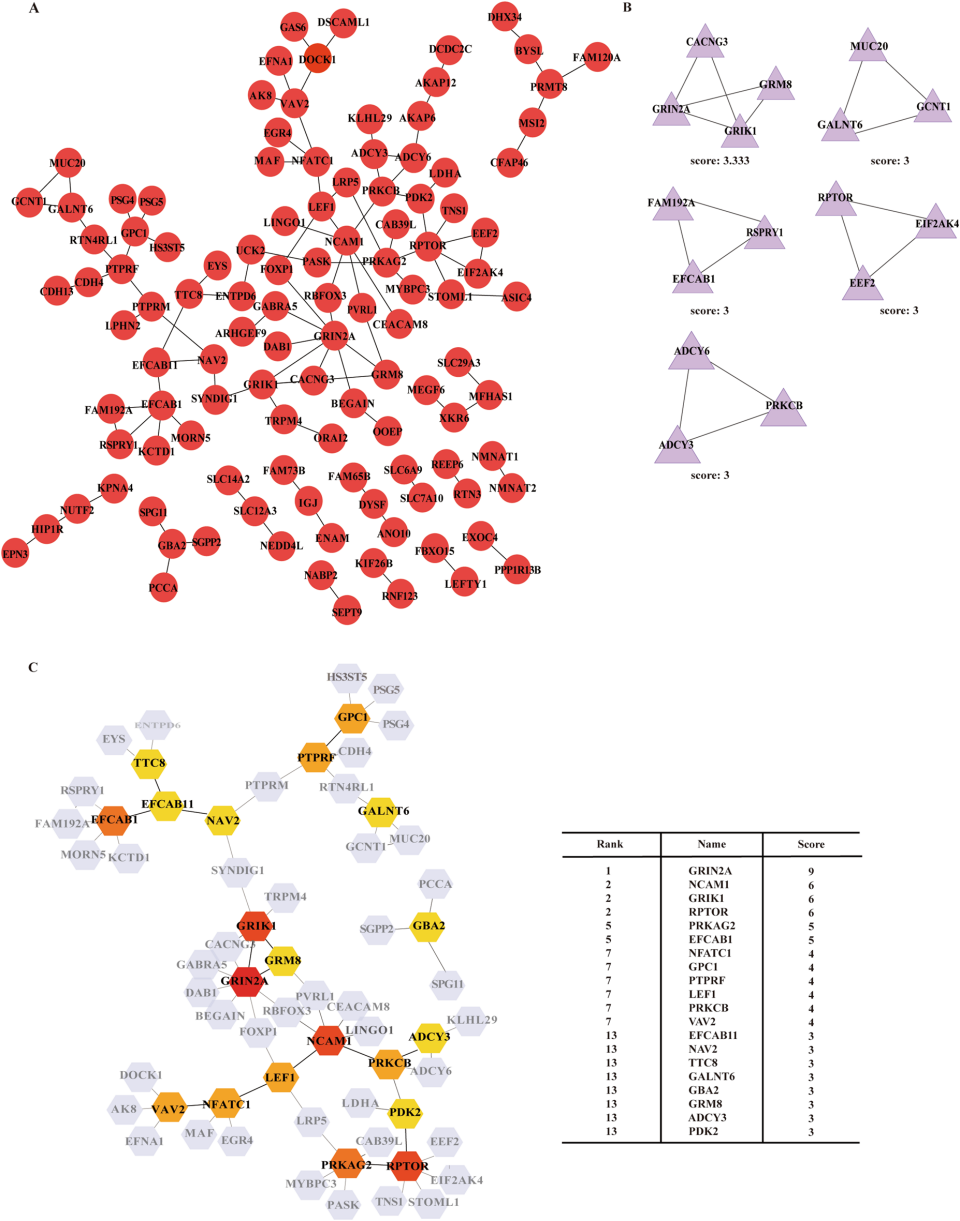
The GGI network between the specifically expressed eccDNA in ESRD. Overview of the GGI network (**A**). Module analysis by MCODE (**B**). The hub genes network (**C**)

To identify the eccDNA that might be crucial in the development of ESRD, we performed computer calculations of the target genes corresponding to these ESRD-specific eccDNA. We built a gene–gene interaction network model of the genes on distinct eccDNAs in the ESRD group using STRING database (Fig. 4A). Subsequently, we uncovered the closely-tied group within the network using the MCODE plugins in Cytoscape, and two clusters were found (Fig. 4B). Moreover, we analyzed the hub nudes among all the genes using cytoHubba and then identified 20 top hub genes. Among the hub genes, GRIN2A, NCAM1, GRIK1, RPTOR, PRKAG2, and EFCAB1 exhibited the highest scores, that was, the greatest gene-to-gene linkages (Fig. 4C).

**Fig. 5:**
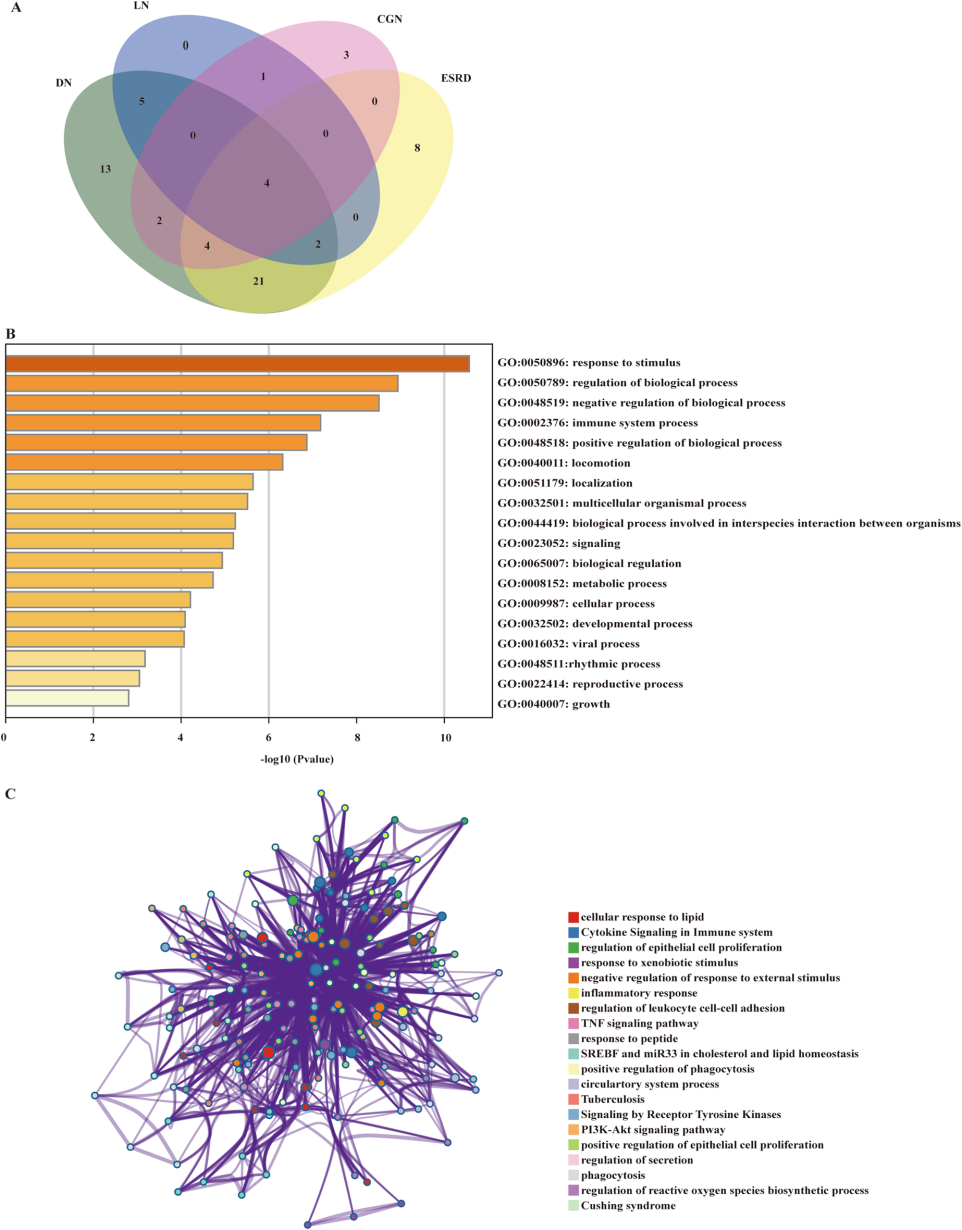
The function of ESRD-related eccDNA-targeting gene. Venn diagram shows the number of overlap target genes of eccDNA and known kidney-related genes in diverse kidney diseases (**A**). The GO biological processes of ESRD-related overlap genes enriched in Metascape (**B**). Pathway and process network analysis of ESRD-related overlap genes (**C**)

After that, we looked for the target genes of ESRD-specific eccDNA that have been reported to link to ESRD, suggesting that their responding eccDNAs might also contribute to ESRD. We searched for the published functional genes of kidney diseases using the phenopedia and DisGeNet databases, and the result showed that a total of 68 genes of eccDNA in ESRD patients were found in the genes known for kidney diseases. The venn diagram exhibited the distribution of 68 genes in various diseases, including 39 genes in ESRD, 51 genes in DN, 14 genes in CGN, and 12 genes in LN (Fig. 5A, Additional file 1: Table S1). Subsequently, we investigated the functions of the 39 ESRD-related genes using Metascape database and found that the principal biological processes that these genes participated in were response to stimulus, regulation of biological process, and negative response of biological process (Fig. 5B). Besides, the pathways analysis highlighted pathways such as cellular response to lipid, cytokine signaling in immune system, and regulation of epithelial cell proliferation (Fig. 5C).

## Discussion

Multiple variables have a role in the development of ESRD because it can be exacerbated by the evolution of a range of kidney illnesses. For starters, renal and systemic inflammation are significant causes of ESRD in patients with kidney diseases [35]. Furthermore, multiple studies have revealed that TFs, DNA, and chromosome damage, as well as metabolites in urine and blood, are all linked to the development of ESRD and can be manifested to be potential diagnostic and prognostic biomarkers [36–38]. However, there are currently no daily clinical examinational biomarkers for ESRD. With advances in technology of sequencing, quantities of latest studies have discovered that eccDNA physically excised from the chromosome is involved in a wide range of biological processes, including cell–cell communication, aging, intercellular genetic heterogeneity, regulating innate immunity, transcribed into noncoding RNAs, and participates in the cancer physiological processes [39–41]. However, the biochemical function of eccDNA in ESRD patients is inadequately defined. As far as we know, this study is the first to illustrate the expression and function of eccDNA in ESRD patients. This study extends the knowledge of the characteristics of eccDNA for ESRD patients. Using the circle-seq technology, we were able to identify eccDNA in PBMCs from ESRD patients and healthy people, as well as reveal the characteristics of eccDNA in PBMCs from patients with ESRD, such as the number of eccDNA, length distribution, genome distribution, motif, and function of genes on eccDNA. Finally, we identified 20 hub genes and 39 ESRD-related genes of ESRD-specific eccDNA-targeting genes.

EccDNA is featured with motifs siding the start and end. Motifs are DNA sequences that provide binding sites for a type of protein called TFs, which govern the activation or repression of gene expression by recognizing motifs found at regulatory regions to regulate downstream chromatin processes [42]. The major ESRD transcription factor GLIS3 in this study, which belongs to the Krüppel-like zinc finger protein family, is mostly expressed in the kidney, thyroid, and pancreas. GLIS3 has been indicated to act a significant part in preserving the natural structure and function of the kidney as part of transcription regulatory networks, and GLIS3 mutant develops polycystic kidney disease [43]. However, whether GLIS3 leads to other kidney diseases even ESRD remains room for further research. Furthermore, the transcription factor Egr2 has been demonstrated to play a role in neutrophil degranulation and immunological activation in ESRD patients on nocturnal hemodialysis [44]. Pitx1, IRF3, and ETS1 are also TFs involved in the pathogenesis of kidney diseases [45–47]. Hence, we conjecture that eccDNA develops ESRD as a result of the TFs mentioned above.

In this work, we discovered 20 top eccDNA hub genes in which NCAM1, NFATC1, PRKCB, LEF1, PRKAG2, and GRM8 were strongly linked to a variety of kidney disorders. For example, gene NFATC1 has been associated with LN [48], gene PRKCB1 has been linked to the progression from DN to ESRD [49], transcription factor LEF1 encoded by gene LEF1 engaged in the Wnt signaling pathway is linked to CKD [50], and gene PRKAG2 is a fresh locus for CKD [51]. In addition, we searched databases and discovered that some ESRD-specific targeting-genes were known to be related to several kidney diseases involved in this study (Additional file 1: Table S1). Obviously, the majority of ESRD-related genes were linked to DN, CGN, or LN as well, which was consistent with the progression of primary diseases into ESRD. Among them, we noticed that CCL2, CCR2, MYH9, and IL10 were present in all four diseases. According to the reports, monocyte chemoattractant protein 1 (MCP-1) encoded by CCL2 was a biomarker in kidney diseases, suggesting kidney damage and inflammation, and CCL2 itself increased in macrophages and was related to renal fibrosis in a renal atrophy model [52]. The genotype frequency of polymorphisms in CCR2 and IL10 showed a great difference between ESRD and controls, especially IL10, which demonstrated their susceptibility to ESRD [53]. MYH9 mutation might disorder renal epithelial transport pathways and further result in kidney diseases [54]. The above ESRD-related genes and functional analysis in Fig. 5C were displayed that inflammation and renal epithelial cell dysfunction were essential mechanisms in ESRD and implied that eccDNA in these genes played a significant part in the progression of ESRD which was the subject of further research.

According to GO analysis, “regulation of cell shape” is the most enriched biological process among the genes predicted by specifically expressed eccDNA in the ESRD group as compared to the NC group. Planar cell polarity (PCP) refers to the coordinated orientation of cells in the tissue plane. Protein encoded by PCP genes and PCP signaling pathway regulate cell shape and behavior, as well as kidney development and diseases, such as polycystic kidney disease and Congenital Anomalies of the Kidney and Urinary Tract (CAKUT) [55]. In addition, actomyosin, a prominent cellular target of the PCP signaling pathway, not only regulates cell shape and motility, but it can also be cleaved by activated caspase-3, causing muscle atrophy in patients with CKD [56]. These findings suggest that eccDNA hub genes may have a role in the pathophysiology of ESRD via regulating cell shape. Furthermore, in comparison to the NC group, "glutamate receptor activity" is the most enriched molecular function of specifically expressed eccDNA in the ESRD group. Ionotropic receptors, such as NMDA receptors, AMPA receptors, and KA receptors, along with metabotropic L-Glu receptors (mGluRs), are the two types of glutamate receptors. The toxicity of overactivated NMDA receptors on renal cells has been demonstrated [57]. Ca^2+^ influx and oxidative stress are caused by sustained NMDA receptors activation, which can contribute to glomerulosclerosis. Ca^2+^ influx pathways TRPC6 that amplifies Ca^2+^ excess activated by NMDA receptors regulates Rac1 of the Rho protein family to modulate signal transduction that influences a great many aspects of cell behavior, including cytoskeletal dynamics in podocytes [58]. The abnormality of actin cytoskeleton then breaches the barrier of proteinuria and finally gives rise to CGN, such as focal segmental glomerulosclerosis (FSGS) [59], which is the most common cause of ESRD and has the most cases in this study, implying that the aforesaid hub genes may be involved in the pathogenic process of ESRD. The primary pathway of ESRD patients with a comparison of healthy individuals in the present study is “phosphatidylinositol signaling system” among the top 20 enrichment KEGG pathways. Phosphatidylinositol signaling pathway is involved in a variety of biological activities, such as cell proliferation, cell differentiation, apoptosis, and membrane trafficking [60]. Among this signaling system, abnormal activation of the phosphoinositide 3-kinase gamma (PI3Kγ) signaling pathway has been shown to play an important role in the regulation of profibrotic phenotypes. In kidney disease, blocking PI3Kγ signaling pathway in Ang-II-induced kidney damage could alleviate renal injury and fibrosis, and thus improve renal functions, as well as be investigated as a fresh therapeutic method for the treatment of renal fibrosis, renal hypertension, and CKD [61]. Furthermore, protein-energy wasting characterized by muscle wasting is obviously manifested in ESRD patients, increasing the morbidity and mortality [62]. A decrease in PI3K activity in skeletal muscle has been shown to aggravate caspase-3 activity and enhance protein degradation, leading to and speeding up wasting through hemodialysis [63]. This latently indicated that eccDNAs from hub genes could be utilized as biomarkers of diagnosis and progression for ESRD. We look forward to further experimental testimonies to validate these findings.

Despite a bit of advances made by relying on genomic and bioinformatics analysis, it is vital to be acknowledged that there are still limitations to current study. Above important, although the discovery of expression of hub genes on eccDNA in the PBMCs of ESRD patients has been observed by sequencing, there is a paucity of further validation. Additionally, their expression and mechanisms have not yet been authenticated by functional experiments in ESRD. In the next part, fewer cases were included and failed to be gathered by a single primary disease.

## Conclusions

Our genomics study revealed the characteristics and specific expression profiles of eccDNA in the PBMCs from ESRD, enabling us to explore the expression and preliminary functions of eccDNA-targeting genes in the pathogenesis of ESRD. Further research is awaited to analyze and prove the significance of eccDNA to the mechanisms of ESRD.

## Supplementary Information

**Additional file 1: Table S1.** The known disease-related genes from specific eccDNA-targeting genes in ESRD group.

## Data Availability

The datasets used and analyzed during the current study are available from the corresponding author on reasonable request.

## Abbreviations

BUN: Blood urea nitrogen
CAKUT: Congenital Anomalies of the Kidney and Urinary Tract
CGN: Chronic glomerulonephritis
CKD: Chronic kidney disease
DAVID: Database for Annotation, Visualization and Integrated Discover
DMs: Double-minutes
DN: Diabetic nephropathy
eccDNA: Extrachromosomal circular DNA
eGFR: Estimated glomerular filtration rate
ESRD: End-stage renal disease
FSGS: Focal segmental glomerulosclerosis
GFR: Glomerular filtration rate
GGI: Gene–gene interaction
GO: Gene Ontology
Hb: Hemoglobin
HTN: Hypertension
KDIGO: Kidney Disease: Improving Global Outcomes
KDOQI: Kidney Disease Outcomes Quality Initiative
KEGG: Kyoto Encyclopedia of Genes and Genomes
KRT: Kidney replacement therapy
LINE: Long interspersed nuclear elements
LN: Lupus nephropathy
LTR: Long terminal repeat
MCODE: Molecular Complex Detection
mGluRs: Metabotropic L-Glu receptors
MT: Mitochondria
mtDNAs: Mitochondrial DNAs
NC: Normal control
PBMC: Peripheral blood mononuclear cell
PCP: Planar cell polarity
PI3Kγ: Phosphatidylinositol 3-kinase gamma
PTH: Parathyroid hormone
rRNA: Ribosomal RNA
Scr: Serum creatinine
scRNA: Single-cell RNA
snRNA: Small nuclear RNA
spcDNAs: Polydispersed circular DNAs
srpRNA: Signal recognition particle RNA
TF: Transcription factor
UCSC: University of California Santa Cruz
