# Supplementary material for: The characteristics of extrachromosomal circular DNA in patients with end-stage renal disease
Source: Eur J Med Res. 2023 Mar 27;28:134. doi: 10.1186/s40001-023-01064-z (PMC10041755; doi:10.1186/s40001-023-01064-z)
Supplement: Supplementary file 1 — Additional file 1: Table S1. The known disease-related genes from specific eccDNA-targeting genes in ESRD group. [file 40001_2023_1064_MOESM1_ESM.docx]

Table S1 The known disease-related genes from specific eccDNA-targeting genes in ESRD group.

| Group | ESRD | DN | CGN | LN |
| --- | --- | --- | --- | --- |
| Gene Name | ABCA1, ADD1, AHR, ALOX5, APOL3, BDKRB2, CCL2, CCR2, CREB1, CNDP1, COL4A3, CXCR2, CYBA, CYP2C9, DPP4, EGFR, ELMO1, ENHO, FTO, HLA-DRB1, IL2RB, IL10, LDLR, LIMK2, MYH9, NAT2, NOD2, PTPN1, PPARA, RNLS, SLC12A3, SLC12A5, SLC13A3, STAT4, TNFRSF1B, TNXB, VDR, VEGFC, XRCC1 | ABCA1, ADORA2B, BDKRB2, CCL2, CCNE1, CCR2, CNDP1, COL4A3, CREB1, CXCR2, CYBA, CYP2C9, DPP4, EGFR, ELMO1, ENHO, EP300, EPAS1, FTO, GRK4, HNF4A, HPSE, HLA-DRB1, IL10, IL19, ITPR1, LDLR, LEF1, LIMK2, MAPK8, MYH9, NAT2, NOD2, PIK3CB, PIK3CD, PPARA, PRKAA2, PTPN1, RELA, SGK1, SLC12A3, STAT4, TNXB, TNFRSF1B, TRPC6, TTC39C, UNC13B, VDR, VEGFC, VTN, XRCC1 | BLK, CCL2, CCR2, ELMO1, EPAS1,  HLA-DRB1, IL10, MYH9, NAT2, PLA2R1, PXK, STAT1, TRPC6, VDR | CCL2, CCR2, EGFR, HNF4A, IL10, MYH9, NOD2, RELA, SGK1, STAT1, PIK3CD, VTN |

ESRD, end-stage renal disease; DN, diabetes nephropathy; CGN, chronic glomerulonephritis; LN, lupus nephritis.
